# Effects of screen-based retinal light stimulation measured with a novel contrast sensitivity test

**DOI:** 10.1371/journal.pone.0254877

**Published:** 2021-07-29

**Authors:** Antonia Neumann, Katharina Breher, Siegfried Wahl

**Affiliations:** 1 Institute for Ophthalmic Research, Eberhard Karls University Tübingen, Tübingen, Germany; 2 Carl Zeiss Vision International GmbH, Aalen, Germany; University of Oxford, UNITED KINGDOM

## Abstract

Myopia is increasing worldwide hence it exists a pressing demand to find effective myopia control strategies. Previous studies have shown that light, spectral composition, spatial frequencies, and contrasts play a critical role in refractive development. The effects of light on multiple retinal processes include growth regulation, but also visual performance and perception. Changes in subjective visual performance can be examined by contrast sensitivity (CS). This study was conducted to investigate whether retinal light stimulation of different wavelength ranges is able to elicit changes in CS and, therefore, may be used for myopia control purposes. In total, 30 right eyes were stimulated with the light of different wavelength ranges, including dominant wavelengths of ∼480 nm, ∼530 nm, ∼630 nm and polychromatic light via a commercial liquid crystal display (LCD) screen. Stimulation was performed screen full-field and on the optic nerve head only. CS was measured before any stimulation and after each stimulation condition using a novel and time-efficient CS test. Post-stimulation CS changes were analyzed by ANOVA regarding the influencing factors spatial frequency, stimulation wavelength and stimulation location. A priorly conducted verification study on a subset of five participants compared the newly developed CS test to a validated CS test. The novel CS test exhibited good reliability of 0.94 logCS and repeatability of 0.13 logCS with a duration of 92 sec ± 17 sec. No clinically critical change between pre- and post-stimulation CS was detected (all p>0.05). However, the results showed that post-stimulation CS differed significantly at 18 cpd after stimulation with polychromatic light from short-wavelength light (p<0.0001). Location of illumination (screen full-field vs. optic nerve head) or any interactions with other factors did not reveal significant influences (all p>0.05). To summarize, a novel CS test measures the relationship between retinal light stimulation and CS. However, using retinal illumination via LCD screens to increase CS is inconclusive.

## Introduction

The prevalence of myopia has been increasing worldwide, and by the year 2050, half of the world population is assumed to be myopic [1, 2]. Myopia, also known as short-sightedness, is a spherical refractive error defined by focusing the light anterior to the retina, either due to an elongated ocular axial length or the excessive refractive power of the eye lens and cornea [1, 3–6]. In addition, myopia presents a risk for the eye health [7, 8], which leads to a need for effective and safe control strategies to inhibit myopia onset and progression [2]. Current myopia control strategies include optical, pharmacological, environmental and surgical options [9, 10]. Moreover, recent studies showed that environmental factors play an important role for refractive development [7, 11–13]. Especially spending time outdoors, under conditions of higher light intensities, higher spatial frequencies, a broader range of contrasts and short- to middle-wavelength sunlight is suggested to prevent myopia onset and slow down myopia progression in humans [12–14].

Along this line, animal models that were exposed to light of different wavelengths indicated inconclusive results regarding axial elongation. In mice [11, 15–19], chicks [20–24] and fish [25, 26] short-wavelength light promote hyperopic shifts, whereas the eyes of rhesus monkeys [27–30] and guinea pigs [11, 30–35] reacted oppositely. Hypotheses on light-induced shifts in refractive error are commonly based on the longitudinal chromatic aberration [36], creating myopic and hyperopic defocus as growth stimulus for the retina [10, 12, 37]. In addition, photoreceptor activation, retinal signaling and neuromodulator activity have an influence on regulation of ocular growth [38]. Especially the neuromodulator dopamine is suggested to be up-regulated by light [39] via activation of melanopsin, a photopigment of intrinsically photosensitive retinal ganglion cells (ipRGCs) [2, 40–42] with a peak spectral sensitivity of ∼482 nm [15, 41–44]. Comparing the distribution of ipRGCs, cones and rods, about 120 million rods, 6 million cones and 50,000 melanopsin containing ipRGCs are distributed around the retina [41]. Melanopsin activation is assumed to enhance cone sensitivity [43, 45].

Therefore, light-induced changes in retinal signaling and visual performace could be detected via psychophysically testing of contrast sensitivity (CS) [46]. This hypothesis is reinforced as reduced CS was found in myopes, being associated with decreased photoreceptor sensitivity and defocus [39, 47].

Myopia control is possible through modulated photoreceptor activation cascades with for example illumination. This purpose was carried out in a previous virtual-reality study using blue light to stimulate different areas of the retina [48]. However, this study aimed to investigate the influence of stimulation via commercial screen technology on a larger sample size. For a broad usage outside laboratory settings a commercially available screen technology for stimulation conditions and assessing CS was used in this current study. For this purpose, a novel, time-efficient, and suitable CS test was developed.

## Materials and methods

### Inclusion and exclusion criteria

This prospective main study and the additional verification study of the novel CS test were carried out at the University of Tübingen. The study protocol followed the Declaration of Helsinki 1964 and the following amendments, and the data protection regulations. The study was approved by the ethics committee of the Faculty of Medicine of the University of Tübingen. Written informed consent was obtained from all subjects prior to the measurements. Participants between 18 years and 40 years of age were included, with a spherical refractive error of ±6.0 D or lower, cylindrical refractive error of maximum −2.0 D, best corrected visual acuity of at least 0.1 logMAR and no known ocular diseases. Additionally, participants with a pupil diameter of less than 4.0 mm were excluded from the main study’s data analysis. To assess inclusion the following tests were conducted prior to the measurement. Color vision was determined with the Ishihara test (Kanehara Trading Inc., Tokyo, Japan). Objective refraction was measured with the ZEISS i.Profiler plus (Carl Zeiss Vision GmbH, Aalen, Germany). The ZEISS VISUSCREEN 500 and VISUPHOR 500 (Carl Zeiss Vision GmbH, Aalen, Germany) were used to assess subjective refraction. Optical coherence tomography was conducted with the ZEISS PlexElite 9000 (Carl Zeiss Meditec Inc., Dublin, USA) to examine eye health. Biometry of the eye was measured by ZEISS IOL Master 700 (Carl Zeiss Meditec AG, Jena, Germany).

### Apparatus and stimulation conditions

All test procedures were based on the software Matlab (MATLAB R2020a, MathWorks Inc., Natick, MA, USA) using the Psychophysics Toolbox Version 3 (PTB) [49, 50]. All stimuli were displayed on a liquid crystal display (LCD) screen (ViewPixx/3D, VPixx Technologies Inc., Saint-Bruno, Canada), which contains the DATAPixx Toolbox. The experiment included in total eight different stimulation conditions including screen full-field (FF) stimulation and only optic nerve head (ONH) stimulation with the following wavelength ranges with expected exposing wavelengths of ∼480 nm (S), ∼530 nm (M), ∼630 nm (L) and ∼380 nm–780 nm (P) [51]. Wavelengths were chosen based on spectral sensitivities of melanopsin containing ipRGCs, middle-wavelength cones and long-wavelength cones, whereby P and FF stimulation conditions were defined as control factors. To achieve a constant luminance of 40 cd/m^2^, the monitor settings were adjusted according to the wavelengths’ spectral transmission.

All measurements were performed on the right eye, while the left eye was occluded. Individual refractive errors were corrected to distance vision using trial lenses (Oculus BK 1/T, Oculus GmbH, Wetzlar, Germany) in a trial frame (Oculus B5, Oculus GmbH, Wetzlar, Germany). An artificial pupil with a diameter of 4 mm [52] was placed in front of the participants’ eye to ensure a constant retinal illumination area and exclude confounding factors for CS measurements between study participants [53]. Therefore, the individual pupil size was recorded before and after the main experiment using an USB-camera (DMK 22AUC03, The Imaging Source, Germany) connected with an infrared light-emitting diode.

### Psychophysical testing procedure of the Adjustment CST

**Fig 1:**
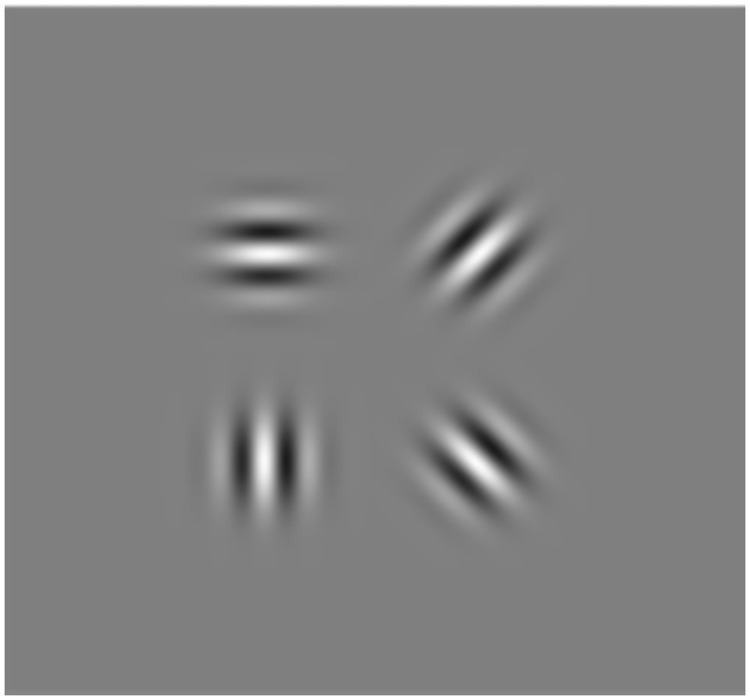
Example of one trial of Adjustment CST consisting of four Gabor patches at four different orientations and a spatial frequency of 6 cpd.

CS was measured with a newly developed, computer-based CS test in a time efficient manner, the so-called Adjustment CST. It is based on the psychophysical method of adjustment, a non-forced choice method measuring contrast thresholds as the mean value over barely visible stimuli starting with contrasts below the expected thresholds [54, 55]. The test incorporated Gabor patches with a visual angle of 1.7° each. The stimulus size was adjusted according to the lens magnification factor of correction lenses to keep a constant retinal image size for all participants [56]. Adjustment CST presents four Gabor patches per trial with equal spatial frequency (SF; measured in cycles per degree (cpd)) but different orientations (0°, 45°, 90° and 135°), see Fig 1.

**Fig 2:**
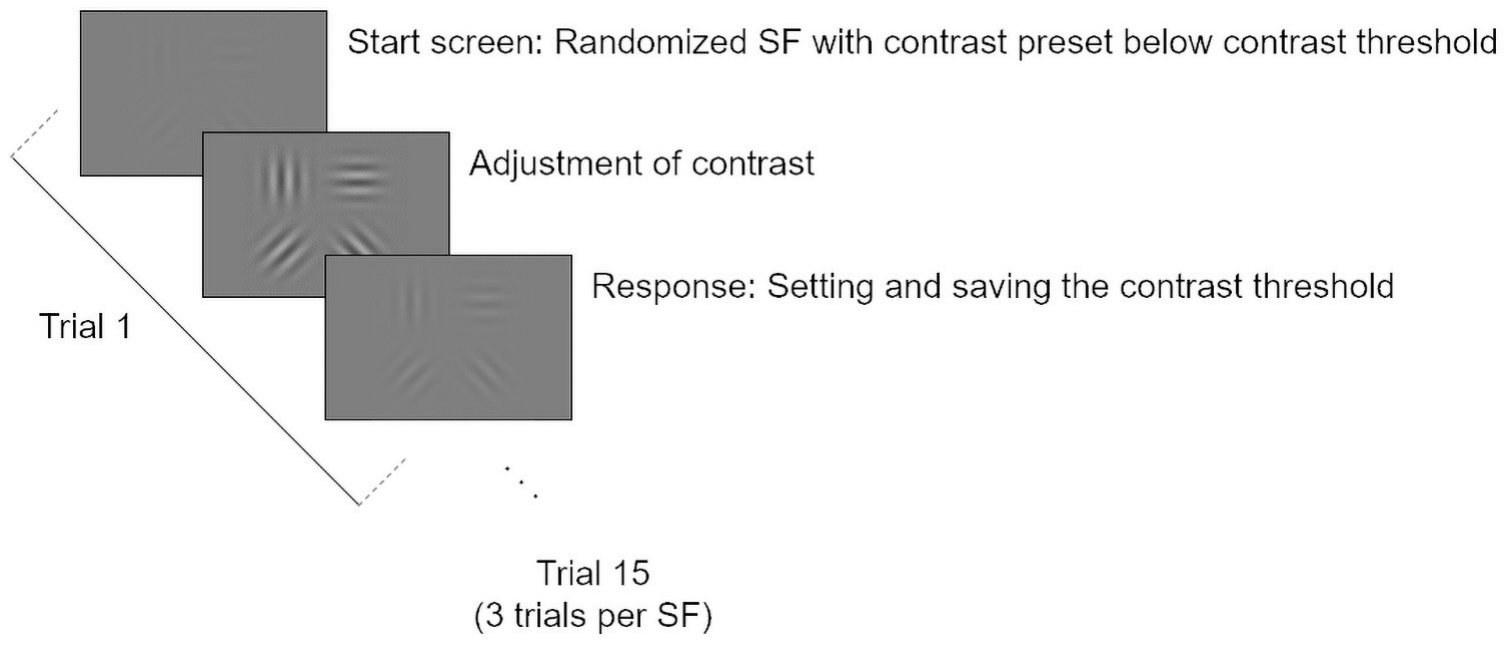
Psychophysical procedure of the new contrast sensitivity (CS) test (Adjustment CST) following the method of adjustment and a total of 15 trials, which means all five selected spatial frequencies were measured three times.

The test includes five randomized presented SFs (3 cpd, 6 cpd, 12 cpd, 18 cpd and 24 cpd), each measured three times. Each trial started with a preset contrast threshold below the CS function. Participants were instructed to adjust the contrast such that all four Gabor patches are just barely visible. Acoustic feedback was provided when the contrast adjustment was set and saved as the contrast threshold. Fig 2 visualizes the test procedure.

### Verification study

The Adjustment CST was verified against a computer-based standardized and validated CS test, the Tuebingen Contrast Sensitivity Test (TueCST) [56]. It is based on a four alternative forced choice (4AFC) method incorporating a Bayesian adaptive staircase procedure, the so-called psi-method. A total of 50 trials per SF was applied as recommended. Each trial presents a Gabor patch with a visual angle of 1.7° in a randomized orientation (0°, 45°, 90° or 135°) for tested SFs of 3 cpd, 6 cpd, 12 cpd, 18 cpd and 24 cpd [56].

Before starting the CS measurements, participants were seated in 1 m distance to the screen in a 25 lux lightened room for 5 min to ensure equal adaptation. After an initial training period, measures by the Adjustment CST and TueCST were performed twice in a randomized order.

### Main study protocol

**Fig 3:**
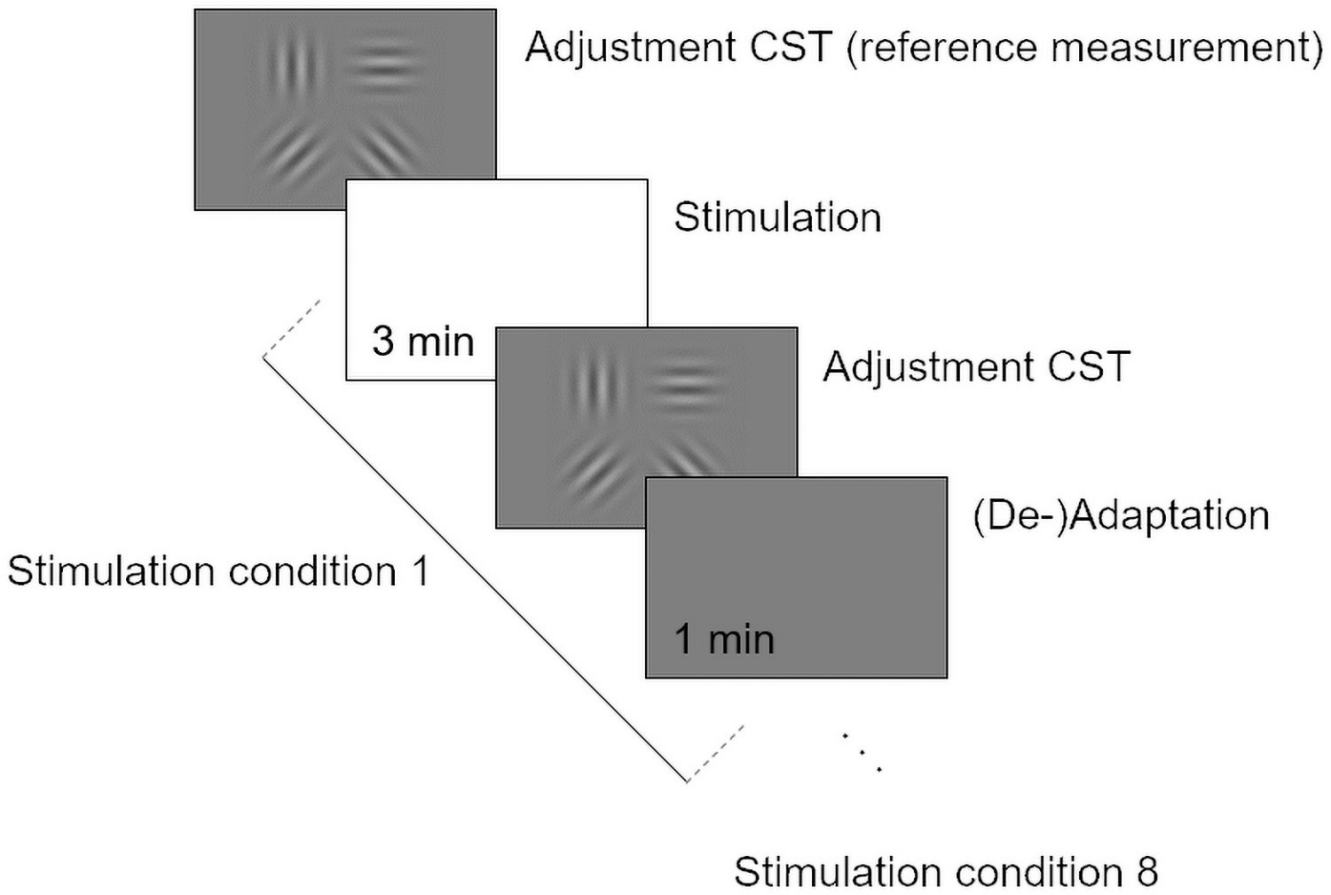
Main study test procedure with a total of 8 repetitions due to 8 stimulation conditions.

Prior to the illumination, the individual ONH sizes and positions were detected. Therefore, Goldmann-perimetry standard stimulus was radially moved from inside the expected ONH (not seen) to outside (seen). The detected ONH was subsequently resized to 70% of the original size to avoid false-positive illumination of the surrounding retina during ONH-only illumination conditions. It was followed by a ten-minute adaptation in a 25-lux lighted room, looking on a gray screen in 1 m distance with a mean luminance of 40 cd/m^2^. Afterward, the right eyes pupil size was recorded. To get familiar with the new Adjustment CST, each participant had to perform training in advance. Thereafter, CS was measured before any illumination (reference CS) and after each stimulation condition. Eight randomized stimulation conditions had to be performed, including four wavelength ranges (S, M, L, and P) and two locations (FF and ONH). Fig 3 consist of the CS test procedure of the main study.

### Statistical analysis

Data analysis was performed using Matlab (MATLAB R2020a, The MathWorks, Inc., Natick, MA, USA) combined with the Statistics and Machine Learning Toolbox. The verification study results were tested regarding intraclass correlation coefficient (ICC) for reliability, Bland-Altman analysis for agreement, and coefficient of repeatability (COR) for repeatability. ICCs smaller than 0.40 are considered as poor, between 0.40 and 0.59 as fair, between 0.60 and 0.74 as good and between 0.75 and 1 as excellent reliability [57].

Normal distribution of the main study results was tested with Shapiro-Wilk test. CS values of the testing conditions against the reference value were compared via the Wilcoxon-Test. The area under the logarithmic contrast sensitivity function (AULCSF) was calculated to compare measurements among logarithmic SFs [58]. A three-factor repeated-measures analysis of variance (ANOVA) was fitted to identify which stimulation conditions significantly affect CS. For the ANOVA, the factors were defined as follows: CS as dependent variable, no between-subject factor, stimulation wavelength (four levels: S vs. M vs. L vs. P), location (two levels: ONH vs. FF) and SF (five levels: 3 cpd, 6 cpd, 12 cpd, 18 cpd, 24 cpd) as the three within-subject factors. Significant main effects and their interactions underwent subsequent posthoc testing with multiple comparisons and Bonferroni correction. Statistical results were considered significant if p<0.05 for a significance level of *α* = 0.05.

## Results

### Baseline values

A total of 30 participants was included in the main study, all with normal color vision. The mean age of the participants was 23.93 years ± 3.88 years. Mean objective and subjective refraction of the right eyes were -1.58 D ± 2.04 D and −1.58 D ± 2.10 D with a visual acuity of at least 0.1 logMAR. The mean eye length was 24.17 mm ± 1.24 mm. Before the main procedure, pupil measurements resulted in pupil size of 6.20 mm ± 1.04 mm and 6.02 mm ± 0.99 mm at the end of the experimental procedure. The averaged time for one test session with the Adjustment CST amounts up to 133 sec ± 56 sec.

For verification of the Adjustment CST, a subset of five participants were included with a mean age of 26.20 years ± 1.79 years. All right eyes had a visual acuity of at least −0.1 logMAR, subjective refraction of 0.35 D ± 0.45 D, and an eye length of 23.65 mm ± 0.99 mm. In the verification study, one of five participants had to be excluded for the SF of 24 cpd due to an invalid measurement (n = 4).

### Verification of method of adjustment test procedure

CS was measured by TueCST and Adjustment CST; both showed a typical CS function curve with a maximum at 3 cpd. Table 1 contains all verification results of two repeated measures of each test, including median and interquartile range (IQR), agreement (Bland Altman), reliability (ICC), repeatability (COR) and time. Regarding agreement, Bland-Altman analysis was conducted and reported higher CS values of TueCST of 0.19 logCS (±0.45 logCS) compared to Adjustment CST. ICC was determined and showed an excellent correlation of 0.94 logCS. The Adjustment CST showed equal or slightly better COR values (0.12 logCS to 0.14 logCS) than the TueCST (0.10 logCS to 0.23 logCS). Regarding time efficiency, TueCST took five times longer (490 sec ± 52 sec) than Adjustment CST (92 sec ± 17 sec).

**Table 1:** Verification values of TueCST, Adjustment CST and Adjustment CST versus TueCST regarding median and interquartile range (IQR), agreement (Bland-Altman), reliability (Intraclass Correleation Coefficient (ICC)) and repeatability (Coefficient of Repeatability (COR)) all measured in logCS and time measured in sec (n = 5, except for 24 cpd: n = 4).

Analysis		TueCST	Adjustment CST
**Median ± IQR (logCS)**	**3 cpd**	2.11 ± 0.13	1.89 ± 0.19
	**6 cpd**	2.04 ± 0.29	1.81 ± 0.19
	**12 cpd**	1.62 ± 0.21	1.48 ± 0.15
	**18 cpd**	1.29 ± 0.20	1.11 ± 0.21
	**24 cpd**	0.95 ± 0.26	0.69 ± 0.20
**Bland Altmann (logCS)** **Mean difference** **[lower bound; upper bound]**		0.19 [-0.26; 0.64]	
**ICC (logCS)** **[lower bound; upper bound]**		0.94 [0.90; 0.97]	
**COR (logCS)**	**3 cpd**	0.10	0.12
	**6 cpd**	0.18	0.13
	**12 cpd**	0.23	0.12
	**18 cpd**	0.18	0.13
	**24 cpd**	0.15	0.14
**Time (sec)**		490 ± 52	92 ± 17

### Relationship of light stimulation and contrast sensitivity

**Fig 4:**
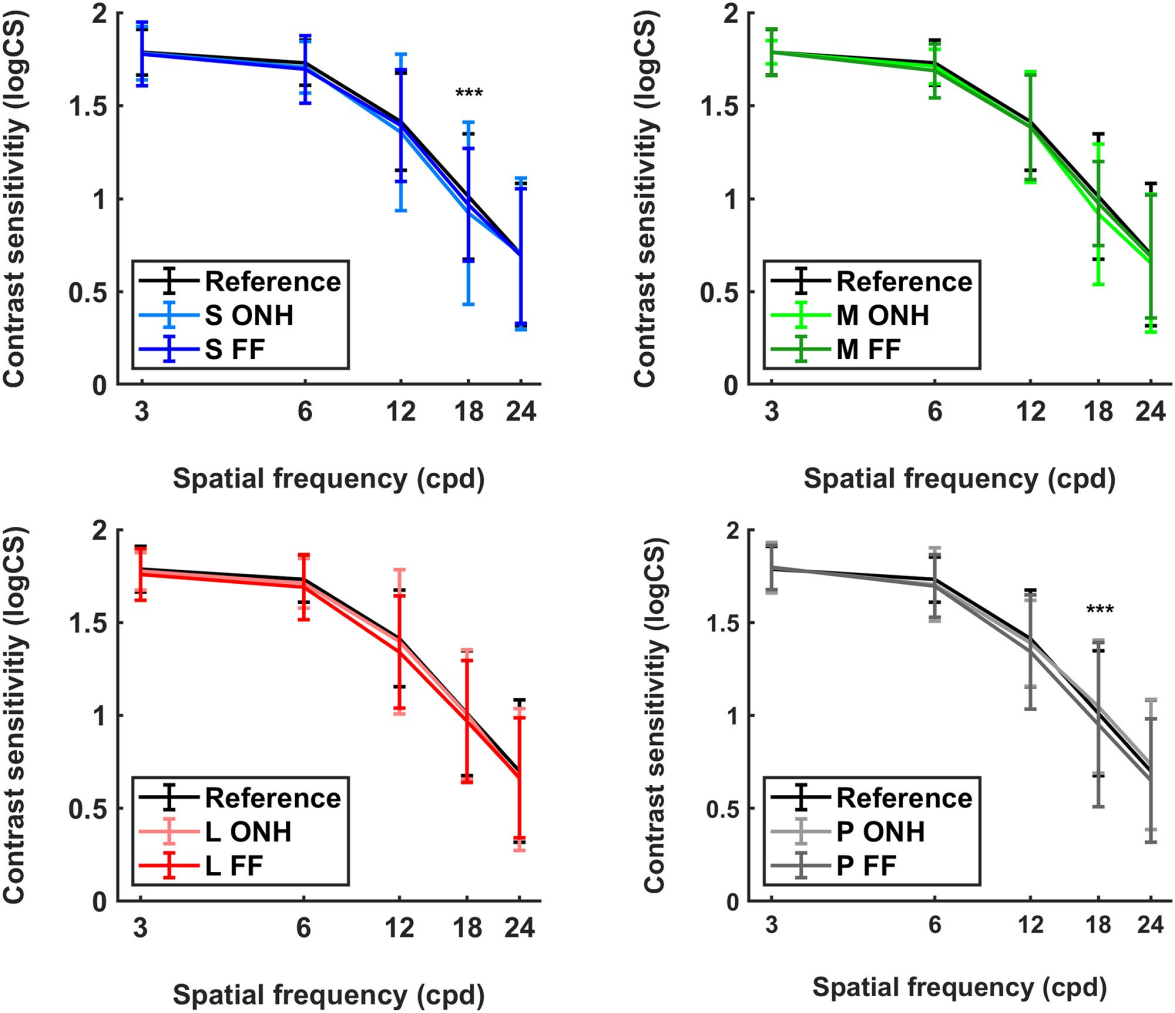
Contrast sensitivity in logCS (median and interquartile range) before (Reference) and after stimulation under different conditions: S ONH (short-wavelength range at optic nerve head), S FF (short-wavelength range via screen full-field), M ONH (middle-wavelength range at optic nerve head), M FF (middle-wavelength range via screen full-field), L ONH (long-wavelength range at optic nerve head), L FF (long-wavelength range via screen full-field), P ONH (polychromatic light at optic nerve head) and P FF (polychromatic light via screen full-field) (n = 30; *** p<0.0001).

The influence of SFs, stimulation wavelengths and stimulation location on CS was investigated in the main study. CS results are listed in Table 2 and represented in Fig 4. There was no statistically significant difference between the reference CS and post-stimulation CS (all p>0.05).

**Table 2:** Contrast sensitivity (CS), in logCS, measured before any stimulation as reference and after each stimulation condition including short- (S), middle- (M), long-wavelength light (L) and polychromatic light (P) stimulating the eye screen full-field (FF) or only the optic nerve head (ONH). Median, interquartile range (IQR) and area under the logarithmic contrast sensitivity function (AULCSF) are listed to compare each condition (n = 30; *** p<0.0001).

Stimulation condition	3 cpd (logCS)	6 cpd (logCS)	12 cpd (logCS)	18 cpd (logCS)	24 cpd (logCS)	AULCSF (logCS)
Reference	1.79 ± 0.12	1.73 ± 0.11	1.41 ± 0.25	1.01 ± 0.31	0.70 ± 0.35	1.32
S-ONH	1.78 ± 0.14	1.71 ± 0.13	1.36 ± 0.38	0.92 ± 0.49 ***	0.70 ± 0.40	1.29
S-FF	1.78 ± 0.16	1.69 ± 0.16	1.39 ± 0.29	0.97 ± 0.29 ***	0.69 ± 0.34	1.30
M-ONH	1.79 ± 0.06	1.71 ± 0.09	1.39 ± 0.28	0.92 ± 0.36	0.65 ± 0.35	1.29
M-FF	1.79 ± 0.12	1.69 ± 0.14	1.38 ± 0.27	0.97 ± 0.22	0.69 ± 0.33	1.30
L-ONH	1.78 ± 0.09	1.71 ± 0.12	1.40 ± 0.36	1.00 ± 0.32	0.66 ± 0.37	1.31
L-FF	1.76 ± 0.13	1.69 ± 0.17	1.34 ± 0.30	0.97 ± 0.32	0.66 ± 0.30	1.28
P-ONH	1.79 ± 0.13	1.70 ± 0.18	1.39 ± 0.22	1.05 ± 0.35 ***	0.74 ± 0.34	1.32
P-FF	1.80 ± 0.12	1.70 ± 0.16	1.34 ± 0.29	0.95 ± 0.42 ***	0.65 ± 0.32	1.28
Median ± IQR	1.79 ± 0.12	1.70 ± 0.14	1.38 ± 0.31	0.97 ± 0.36	0.68 ± 0.35	

When only post-stimulation CS values were analyzed, factor SF showed a significant influence (F = 305.10, p = 3.81e-63), as expected from the regular CS function. Stimulation location revealed no significant effect (F = 2.25, p = 0.14). Wavelengths did not differ significantly from each other (F = 1.25, p = 0.30). In addition, SFs interacted with stimulation wavelength turned out to has a significant effect on CS (F = 1.97, p = 0.03). Therefore, post-hoc analysis was conducted, which revealed one significant effect (p<0.0001) at 18 cpd when comparing S and P stimulation condition (difference = ∣0.05∣, p = 0.004). CS at 18 cpd is higher after stimulation with P than after stimulation with S (Table 2). Interactions of SF and location (F = 1.36, p = 0.25), wavelength and location (F = 1.04, p = 0.38), SF, wavelength and location (F = 0.33, p = 0.97) turned out to be not significant (all p>0.05). Furthermore, analysis revealed in no significant correlation between CS and the degree of myopia, as the between-subject factor of the refractive groups myope and non-myope has been included (p = 0.09).

## Discussion

The purpose of this study was to investigate whether retinal stimulation with light of different dominant wavelengths, emitted by commercially available screen technology, increases the CS using a newly developed CS testing procedure. Two main outcomes can be reported: Firstly, the adjustment-based CS test can be considered as a time-efficient, accurate and repeatable test procedure. Secondly, the interaction of wavelength range and SF has an influence on CS at 18 cpd after P and S stimulation only. However, stimulus location did not show significant effects in this study.

### Verification of Adjustment CST

The Adjustment CST applies the method of adjustment as a non-forced choice method and was designed to measure CS in a time-efficient, reliable and repeatable fashion.

The novel CS test showed excellent reliability, confirmed by high ICC values and good agreement. This reliability approves that the test measures CS in a correct range. However, the TueCST showed higher contrast thresholds than the Adjustment CST. The setup as cause of these biases can be ruled out as the exact same setup was used. Instead, the method of adjustment as psychophysical procedure could be responsible as it is more criterion-based and time unlimited compared to a 4AFC method. Compared to previous data, the method of adjustment is the simplest and most direct method for contrast estimation [59], but lower results are expected using this method [60]. Due to adaptation effects, the adjustment method is not expected to be as precise as forced-choice procedures [61]. Furthermore, increasing contrast from a sub-threshold preset value avoids adaptation compared to supra-threshold starting points, however, still leading to a small amount of contrast adaptation during the adjustment phase [61].

In addition, the Adjustment CST revealed good repeatability with slightly better COR values compared to the TueCST and TueCST validation study [56] while Adjustment CST maintaining high time efficiency. A short duration of contrast testing was among the main priorities for the development of the Adjustment CST, as any post-illumination effects could have been lost due to a lengthy test duration. Furthermore, long and repetitive testing procedures lower concentration and attention [56, 62]. A short test duration in the current study was achieved by simultaneously presenting Gabor patches in four different orientations, which cuts down the required number of trials. Comparing both tests, the TueCST takes about five times longer than the Adjustment CST [56].

### Retinal light stimulation with commercial screen technologies

**Fig 5:**
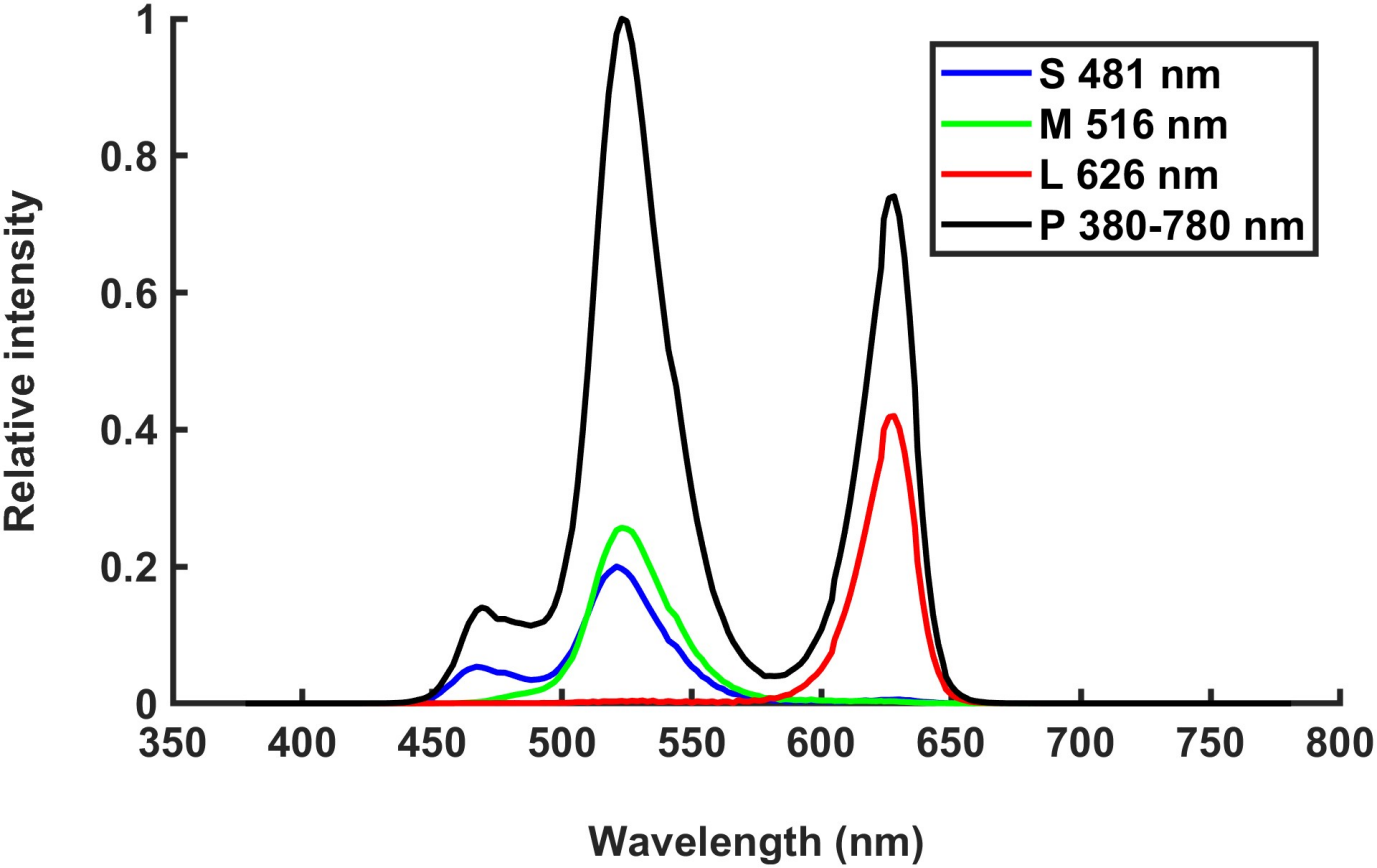
Normalized intensity (normalized spectra including luminosity function V(λ)) of stimulation conditions with short- (S), middle- (M), long-wavelengths (L) and polychromatic (P) light emitted from ViewPixx/3D.

Retinal light stimulation was performed with a commercially available LCD screen technology. With this, it is not possible to emit light of one wavelength but rather parts of the visible spectrum with a dominant wavelength. The chromatic monitor settings were chosen to match the sensitivities of melanopsin-ipRGCs, middle- and long-wavelength cones. This resulted in the following dominant wavelengths [minimum wavelength; maximum wavelength; absolute bandwidth]: 481 nm [444 nm; 572 nm; 128 nm], 516 nm [467 nm; 582 nm; 115 nm], 626 nm [584 nm; 656 nm; 72 nm] and polychromatic [440 nm; 663 nm; 223 nm]. However, these are not narrow-width filter but dominant wavelengths of the spectrum emitted by the screen. Fig 5 consists of normalized intensities, including spectra and luminosity function V(λ) emitted from LCD monitor.

In order to rule out overlapping stimulation effects, a 1 min (de-)adaptation phase was implemented before any light stimulation. This phase leads to at least 75% compensation of chromatic adaptation [61]. Ideally, additional CS measurements right before each stimulation condition would have excluded the effects of chromatic adaptation to previous illumination conditions. This was not done due to doubling the total experimental time and subsequent loss of attention by participants with less reliable results [56, 62].

### Retinal light stimulation affects contrast sensitivity in the context of emmetropization

Higher SFs are known to play a role in emmetropization [24], therefore a range from 3 cpd to 24 cpd were included in the current study to evaluate if there are effects in lower, middle, or higher frequencies. In this study 18 cpd as a higher SF turned out to significantly affect CS after stimulation of polychromatic and short-wavelength stimulation. Whether 18 cpd is the most effective SF regarding emmetropization cannot be concluded from this study. Nevertheless, a recent study on humans found a significant change of CS for the SFs of 6 cpd, 12 cpd and 18 cpd after illuminating the ONH with short-wavelength light [40]. Through photoreceptor activation their sensitivity should be increased which leads to an increased CS. In the current study, we used stimulation via a LCD screen. Post-hoc analysis showed a higher CS after P than after S stimulation. Moreover, another study showed reduced CS in myopes when using a S-cone stimulus [63]. The usage of different methodology could account for the differences in the result. Besides the experimental setup, the photoreceptors’ spectral sensitivity could be a further possible explanation. While S stimulation activates theoretically mainly ipRGCs due their peak spectral sensitivity, P stimulation activates beside ipRGCs all cones [64]. A directly and specifically stimulation of individual photoreceptor classes is not possible using stimulation via commercial screen technologies due to its emission bandwidth. Another reason for the shown significant effect might be a statistical artifact, even possible due to the highest IQR at 18 cpd compared to all other SFs and the fact that ipRGCs do not respond to higher frequencies. Assuming, that a larger reaction of only a few participants led to the given statistical result. In addition, there was no effect found at the higher frequency of 24 cpd. However, this finding would reflect less ipRGC sensitivity and thus less melanopsin expression in S than P condition which seems counter-intuitive.

Another finding was that stimulation location, wavelength range, and their interaction did not significantly affect CS. This finding is somewhat surprising as CS changes would have been expected with the ONH condition due to the high relative density of ipRGCs axons [3, 41, 65]. This finding could potentially be explained by diffuse reflections of light from the targeted ONH across the inner eyeball [66] or the insufficient delimitation of the ONH. Scattering and reflections were not controlled in the current study based on the aim to use a commercially available setup. For FF stimulation conditions, an artificial pupil with a constant diameter was used to minimize this aspect between participants and rule out varying area sizes of illumination [67]. Additional pupil diameter measures were conducted to ensure a pupil size equal to or larger than the artificial pupil diameter.

A critical change of at least 0.2 logCS in absolute differences [56] between pre- and post-stimulation with different wavelength ranges was not found in this study. The fact that only adults were included in the current study could be the reason that there are no significant differences in CS between the different stimulation conditions. Adults commonly have completed eye development, suggesting a stable dopamine level (except circadian rhythms and dependent changes in refractive error, axial length, and retinal dopamine [68–70]). Based on refractive status, in the current study no significant difference in CS was found between myopes and non-myopes. However, there were myopes included with a low- to mid-range of myopia but no high myopes participated in the study. However, it requires further investigation if there indeed was no specific photoreceptor class activation or if the effects were too subtle to be detected with the current setup.

### Influence of different wavelength ranges in context of myopia control

Previous studies showed that stimulation with light and emmetropization have two possible underlying mechanisms. First, short-wavelength light leads to melanopsin activation and increases dopamine [71]. Second, short-wavelength light is focused in front of the retina because of longitudinal chromatic aberration, leading to myopic defocus [36].

Studies on mice [15, 17–19] and chicken [20, 22, 23] have proven that melanopsin activation depends on the retinal dopamine level and thus an impact in modulating vision [11], including CS, visual acuity and light adaptation and axial elongation. [11, 69]. However, findings on rhesus monkey-studies [27, 29, 32] and guinea pig-studies [31–35] are controversial.

In addition, emmetropization might not only be caused by increased photoreceptor sensitivity but also due to focusing short-wavelength light in front of the retina based on longitudinal chromatic aberration and induced monochromatic myopic defocus [36]. A myopic defocus appears protective against myopia progression in human and animal studies [21, 34, 37, 47, 72–75]. The complexity around short-wavelength retinal light stimulation or stimulation with other wavelengths and myopia is high. Therefore, further research is needed to gain understanding how retinal light stimulation affects CS.

## Conclusion

The novel Adjustment CST is able to determine CS in a time-efficient and repeatable fashion. A clinically critical increase of CS after retinal stimulation with light of differing wavelength ranges was not found, neither compared to reference CS without any stimulation nor compared to post-stimulation CS, except the significant effect of P and S stimulation at 18 cpd. Further research is required to deepen the understanding of retinal signaling based on stimulation with different wavelength ranges and translate it into effective, non-invasive strategies to control myopia psychophysically.
